# Prediction of clinical pharmacokinetics of AMG 181, a human anti-*α*_4_*β*_7_ monoclonal antibody for treating inflammatory bowel diseases

**DOI:** 10.1002/prp2.98

**Published:** 2014-12-09

**Authors:** Hong Li, Kathleen Köck, John A Wisler, William A Rees, Peter J Prince, Kai O Reynhardt, Hailing Hsu, Zhigang Yu, Dominic C Borie, David H Salinger, Wei-Jian Pan

**Affiliations:** 1 Pharmacokinetics and Drug Metabolism, Amgen Inc.Seattle, Washington; 2 Comparative Biology and Safety Sciences, Amgen Inc.Thousand Oaks, California; 3 Medical Sciences, Amgen Inc.Seattle, Washington; 4 Inflammation Discovery Research, Amgen Inc.Thousand Oaks, California; 5 Medical Sciences, Amgen Inc.Thousand Oaks, California; 6 Global Development, Amgen Inc.South San Francisco, California

**Keywords:** AMG 181, anti-*α*_4_*β*_7_, cell trafficking, Crohn’s disease, gut homing, human antibody, inflammatory bowel diseases, PK/PD predictions, T cells, ulcerative colitis, *α*_4_*β*_7_ integrin

## Abstract

The purpose of this study was to predict a safe starting dose of AMG 181, a human anti-*α*_4_*β*_7_ antibody for treating inflammatory bowel diseases, based on cynomolgus monkey pharmacokinetic (PK) and pharmacodynamic (PD) data. A two-compartment model with parallel linear and target-mediated drug disposition for AMG 181 PK in cynomolgus monkey was developed. The estimated parameters were allometrically scaled to predict human PK. An *E*_max_ PD model was used to relate AMG 181 concentration and free *α*_4_*β*_7_ receptor data in cynomolgus monkey. AMG 181 clinical doses were selected based on observed exposures at the no adverse effect level of 80 mg·kg^−1^ in monkeys, the predicted human exposures, and AMG 181 concentration expected to produce greater than 50% *α*_4_*β*_7_ receptor occupancy in humans. The predicted human AMG 181 clearance and central volume of distribution were 144 mL·day^−1^ and 2900 mL, respectively. The estimated EC_50_ for free *α*_4_*β*_7_ receptor was 14 ng·mL^−1^. At the 0.7 mg starting dose in humans, the predicted exposure margins were greater than 490,000 and AMG 181 concentrations were predicted to only briefly cover the free *α*_4_*β*_7_ receptor EC_10_. Predictions for both *C*_max_ and AUC matched with those observed in the first-in-human study within the 7 mg subcutaneous to 420 mg intravenous dose range. The developed model aided in selection of a safe starting dose and a pharmacological relevant dose escalation strategy for testing of AMG 181 in humans. The clinically observed human AMG 181 PK data validated the modeling approach based on cynomolgus monkey data alone.

## Introduction

Crohn’s disease (CD) and ulcerative colitis (UC) are the two major types of inflammatory bowel diseases (IBD). Currently, anti-tumor necrosis factor agents (anti-TNFs) are the main class of approved biologics for treating CD and UC. However, there is a need for development of new therapeutics for those patients who do not respond or have developed tolerance to anti-TNFs after initial treatment. Therapies that block the influx of pro-inflammatory cells into the intestinal mucosa through integrin-mediated leukocyte tethering, rolling, and arrest (Hynes 2002) have gained attention in recent years.

Preclinical animal models have demonstrated the roles and benefits of blocking lymphocyte intestinal homing through antagonism of integrin *α*_4_*β*_7_ (Hesterberg et al. 1996; Fedyk et al. 2012; Pan et al. 2012), integrin *β*_7_ (Wagner et al. 1996; Apostolaki et al. 2008; Stefanich et al. 2011), and integrin *α*_4_*β*_7_ ligand MAdCAM-1 (Picarella et al. 1997; Pullen et al. 2009). There are currently several IBD treatments targeting leukocyte migration and adhesion in clinical development, including vedolizumab (anti-*α*_4_*β*_7_), etrolizumab (anti-*β*_7_), PF-00547659 (anti-MAdCAM-1), GSK-1605786 (anti-CCR9), and AJM300 (anti-*α*_4_) (Thomas and Baumgart 2012; Lobaton et al. 2014). Natalizumab (anti-*α*_4_) has been approved in the United States for the treatment of CD (Sandborn et al. 2005; Targan et al. 2007) and vedolizumab has shown clinical benefits in treating both CD and UC (Feagan et al. 2005, 2008; Parikh et al. 2011).

Natalizumab is a humanized immunoglobulin (Ig) G_4_ antibody targeting *α*_4_ and is thus antagonistic against both *α*_4_*β*_1_ for treating relapsing and remitting forms of multiple sclerosis (Polman et al. 2006) and *α*_4_*β*_7_ for treating CD (Sandborn et al. 2005). Natalizumab-mediated inhibition of trafficking of *α*_4_*β*_1_-expressing leukocyte to the central nervous system through pan-*α*_4_ inhibition may be linked to progressive multifocal leukoencephalopathy (PML) caused by reactivation of latent human John Cunningham (JC) polyomavirus infection (Berger and Koralnik 2005). Additionally, natalizumab as well as vedolizumab (IgG_1_) are humanized antibodies and have shown greater than 10% immunogenicity in IBD patients leading to diminishing clinical efficacy (Feagan et al. 2005, 2008; Sandborn et al. 2005; Parikh et al. 2011).

AMG 181, a human monoclonal IgG_2_ antibody that binds specifically to the *α*_4_*β*_7_ integrin, has been designed to reduce or eliminate the immunogenic response and also avoids targeting the *α*_4_*β*_1_-expressing leukocytes implicated in the occurrence of PML reported previously in natalizumab-treated patients. Comprehensive investigational results from AMG 181 in vitro pharmacology experiments and in vivo pharmacokinetic and toxicology studies have been published previously (Pan et al. 2013).

Several recent publications have summarized methods for the first-in-human (FIH) dose estimation (Gibbs 2010; Chen et al. 2012; Zou et al. 2012). Here, a quantitative translation of AMG 181 in vitro and in vivo pharmacology from cynomolgus monkeys to humans is presented that uses 70% and 90% levels of *α*_4_*β*_7_ receptor occupancy to guide the selection of a safe starting dose and pharmacologically relevant single and multiple dose escalation schemes for Phase 1 clinical trials in healthy volunteers and IBD subjects (http://www.clinicaltrials.gov, study identifiers NCT01164904 and NCT01290042). Comparison with the clinical PK from the single-dose Phase 1 study (Pan et al. 2014) validated our modeling predictions.

## Materials and Methods

### In vitro pharmacology studies

AMG 181 (∼144 kD) was manufactured at Amgen Inc. (Thousand Oaks, CA) by expression in a Chinese hamster ovary (CHO) cell line (Hsu et al. 2010). Results from in vitro pharmacology studies including *α*_4_*β*_7_ target receptor occupancy on CD4^+^ T-cell subsets, including central memory, effector memory, and naïve T, have been reported previously (Pan et al. 2013).

### In vivo studies in cynomolgus monkeys

Male and female cynomolgus monkeys (*Macaca fascicularis*), ranging from 2.1 to 6.4 kg, were cared for in accordance with the Guide for the Care and Use of Laboratory Animals, 8th Edition (National Research Council US 2011). Animals were socially housed at an indoor, AAALAC, Intl-accredited facility in species-specific housing. All research protocols were approved by the Institutional Animal Care and Use Committee. Animals were fed a certified pelleted primate diet (PMI #5048, Richmond, IN) daily in amounts appropriate for the age and size of the animals, and had ad libitum access to water (municipality tap water processed through a reverse osmosis filter and passed through ultraviolet light treatment) via automatic watering system/water bottle. Animals were maintained on a 12:12 h light:dark cycle in rooms at 18–29°C (30–70% humidity) and had access to enrichment opportunities (small bits of fruit, cereal, or other treats). All animals were negative for simian retrovirus.

Cynomolgus monkeys were assigned to treatment groups for two single-dose PK/PD Studies A and C, a two-dose (2-weekly doses) toxicology Study B, a 3-month (13-weekly doses; Study D), and a 6-month (24-weekly doses; Study E) Good Laboratory Practice (GLP) toxicology study (Table 1). Pre- and postdose blood samples were collected for PK/PD and immunogenicity assessments. Postdose observations in toxicology studies were conducted to assess the safety of AMG 181. PK samples from the PK/PD studies and the non-GLP toxicology study were analyzed for AMG 181 using an electrochemiluminescence (ECL) immunoassay with a lower limit of quantification (LLOQ) of 2 ng·mL^−1^. The GLP toxicology study used an ECL immunoassay with a LLOQ of 20 ng·mL^−1^.

**Table 1:** Studies in cynomolgus monkeys used for modeling

Study	Description	N	Groups	Treatments
A^1^	Single-dose PK/PD	18 males	6 groups; 3/group	Vehicle control IV; 0.01, 0.1, 0.3, and 3 mg/kg AMG 181 IV; 3 mg/kg AMG 181 SC
B^1^	Two-dose Non-GLP Toxicology	12 males 12 females	3 groups; 4/sex/group	Vehicle control SC QW; 0.5 and 80 mg/kg AMG 181 SC QW
C^1^	Single-dose PK/PD	18 males	3 groups; 6/group	Vehicle control IV; 9 mg/kg AMG 181 IV; 9 mg/kg AMG 181 SC
D^1^	Three-month GLP Toxicology	30 males 30 females	5 groups; 6/sex/group	Vehicle control SC QW; 5, 20, or 80 mg/kg SC QW; 80 mg/kg IV QW
E	Six-month GLP Toxicology	22 males 22 females	3 groups; 6-8/sex/group	Vehicle control SC QW; 20 or 80 mg/kg SC QW

IV, intravenous; SC, subcutaneous; PK, pharmacokinetics; PD, pharmacodynamics; TK, toxicokinetics; QW, once-weekly.

1 PK, results for these studies have previously been reported in Pan et al. (2013).

Serum samples were analyzed for anti-AMG 181-specific antibodies (antidrug antibodies; ADAs) using a validated immunoassay with a lower limit of reliable detection (LLRD) of 20 ng·mL^−1^ rabbit anti-AMG 181 polyclonal antibody in neat pooled cynomolgus monkey serum. Samples with values greater than the predefined assay-specific cut point ranging from 1.08 to 1.14 based on the signal-to-noise ratio (fold difference in the anti-AMG 181-specific antibodies assay signal of samples or positive controls relative to the assay background assessed by the negative control) were defined as ADA positive.

### AMG 181 PK data for modeling

PK data were collected from a total of 119 monkeys (73 males and 46 females) actively treated with AMG 181 for up to 420 days after the first dose, with 86 receiving SC and 33 receiving IV dose(s). The median body weight of the monkeys was 2.8 kg (mean: 3.43 kg; range: 2.1–6.4 kg). In all five studies, anti-AMG 181 antibodies or immunogenic response was observed in a majority of the monkeys, except for those dosed 80 mg·kg^−1^ SC or IV. In many cases, the presence of anti-AMG 181 antibodies clearly impacted the PK. Data point exclusion was based on a two-step process: In a first step, candidates for exclusion were identified based on ADA positivity. In the second step, only data points where PK was also visually impacted were excluded (Fig. S2). This approach resulted in removal of 120 out of the 1743 (6.88%) available PK data points. The remaining 1623 AMG 181 concentration–time values were used for compartmental PK modeling.

### Compartmental modeling of AMG 181 PK data

#### PK structural models

**Figure 1:**
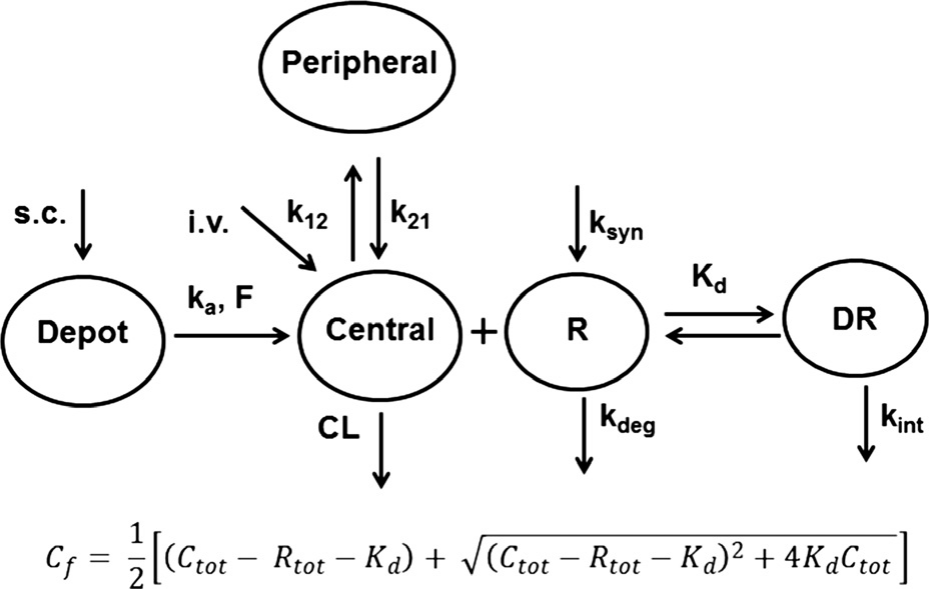
Schema of the two-compartment PK model with target-mediated drug disposition (TMDD) and quasi-equilibrium (QE) approximation.

Visual inspection of the individual cynomolgus monkey PK data showed an initial distribution phase after IV and a terminal nonlinear elimination phase after both IV and SC administration. Individual PK data were simultaneously fit to a two-compartment PK model with target-mediated drug disposition (TMDD) (Mager and Jusko 2001) using quasi-steady-state (QSS) approximation (Gibiansky et al. 2008) or quasi-equilibrium (QE) approximation (Mager and Krzyzanski 2005). A two-compartment PK model with parallel linear and nonlinear Michaelis–Menten (M-M) was also fitted, but this model was not found superior to the simplified TMDD models (data for model comparison on file). For the high multiple doses of 20–80 mg·kg^−1^, which are especially relevant for prediction of clinically relevant doses, the M-M model did not predict the terminal phase as well as the TMDD model. The TMDD model parameters included: the first-order SC absorption rate constant (*K*_A_), central volume of distribution (*V*_c_), linear clearance (CL), the distribution rate constants between the central and peripheral compartments K_12_ and K_21_, the total *α*_4_*β*_7_ receptor concentration (*R*_tot_), the internalization rate constant of drug–receptor complex (*K*_int_), and the QSS constant *K*_ss_ = (*k*_int_ + *k*_off_)/*k*_on_, or the dissociation constant *K*_d_ = *k*_*off*_/*k*_on_ for QE model (Fig.1). TMDD QSS/QE modeling was conducted on molar transformed AMG 181 concentration data with the free AMG 181 concentration (*C*_f_) calculated using the equation shown in Figure 1. The *K*_d_ was fixed at 0.013 nmol/L, the EC_50_ value of AMG 181 binding to monkey primary T cells in vitro.

#### PK variability models

Values for interindividual variability (IIV) of *K*_A_, CL, *V*_c_, and *R*_tot_ were estimated assuming lognormal distribution. The residual variability was assumed to have a mixture of proportional plus additive distribution.

#### Estimation method, diagnosis, and validation

PK datasets and results were constructed, tabulated, and plotted using S-PLUS® (Version 8.2, November 2010; TIBCO Software Inc., Palo Alto, CA) or SigmaPlot (Version 12.5; Systat Software, Inc., Chicago, IL, USA). Modeling of the monkey PK data and simulation of the human PK profiles were performed using nonlinear mixed-effects modeling using NONMEM®, Version 7.2 with gfortran FORTRAN compiler (Beal et al. 2009).

The first-order conditional estimation method with interaction (FOCE INTERACTION) was used to estimate the population PK parameters. Importance sampling method, with EONLY = 1 (“evaluation only”), was applied to facilitate obtaining the standard error of model parameters.

Model selection was guided by visual inspection of goodness-of-fit plots, parameter estimate precision/plausibility, and comparison of objective function values. All parameter estimates were reported with the relative standard error of the estimates (%SE). Internal model validation was carried out by visual predictive check (VPC) plots; four hundred datasets were simulated based on final parameter estimates and the estimated 10th, 50th, and 90th percentiles of the simulated AMG 181 concentration–time profiles were plotted against the observed data.

### AMG 181 PD data and modeling

Study A was a dedicated PK/PD study in cynomolgus monkeys, where AMG 181 occupancy of free *α*_4_*β*_7_ on CD4+ T cell and its subsets were evaluated ex vivo. Mean fluorescence intensity (MFI) readings of free *α*_4_*β*_7_ and the time-matched AMG 181 concentrations in serum were used for curve fitting using an *E*_max_ model:

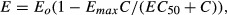


where the effect (*E* or MFI) was estimated through baseline (*E*_0_), maximum effect (*E*_max_), AMG 181 concentration at half-maximal effect (EC_50_), and AMG 181 concentration (*C*). Subsequent calculations for EC_75_, EC_90_, and EC_99_ and the AMG 181 concentrations at 75%, 90%, and 99% maximal effect, respectively, were carried out based on the *E*_max_ model. Results from Studies B–E were not used for PD modeling due to high data variability and limited availability (especially GLP studies).

### Allometric scaling and simulation of human PK

For the prediction and simulation of human PK, the structural PK models developed for cynomolgus monkeys (median observed body weight of 2.8 kg) were assumed to also describe AMG 181 disposition in humans (assumed median body weight of 70 kg) with parameters allometrically scaled (Dong et al. 2011). The AMG 181 CL and *V*_c_ values in human were determined from those in cynomolgus monkey by scaling by body weight ratio with exponents of 0.75 and 1, respectively. The distribution rate constants (K_12_ and K_21_) were scaled with an exponent of −0.25. The *K*_d_ constant was fixed at 0.031 nmol/L, the EC_50_ value of AMG 181 in vitro binding potencies to human primary T cells. Other PK parameters including *K*_A_, F1, *R*_tot_, and *K*_int_ were assumed to be equivalent for cynomolgus monkeys and humans.

### Exposure margins and FIH dose selection

The exposure margins were calculated based on the observed AMG 181 *C*_max_ (4850 *μ*g·mL^−1^) and area under the concentration–time curve (AUC, 485,000 h·*μ*g·mL^−1^) values in cynomolgus monkeys at the no observed adverse effect level (NOAEL; Study D) of 80 mg·kg^−1^ IV divided by the predicted human *C*_max_ and AUC values.

AMG 181 doses for the FIH study were selected based on: the exposure margins; the duration of serum AMG 181 concentration above 50–90% *α*_4_*β*_7_ receptor occupancy; and the maximum recommended starting dose (MRSD) in humans according to the United States Food and Drug Administration (US FDA) guidance (FDA 2005).

### Comparison of predicted versus observed AMG 181 PK in humans

The predicted AMG 181 AUC and *C*_max_ in humans were compared to the AMG 181 PK parameters observed in the FIH study with doses ranging from 7 mg SC to 420 mg IV. Data from the FIH study of AMG 181 have been reported separately (Pan et al. 2014).

## Results

### Compartmental modeling of AMG 181 PK in cynomolgus monkeys

**Figure 2:**
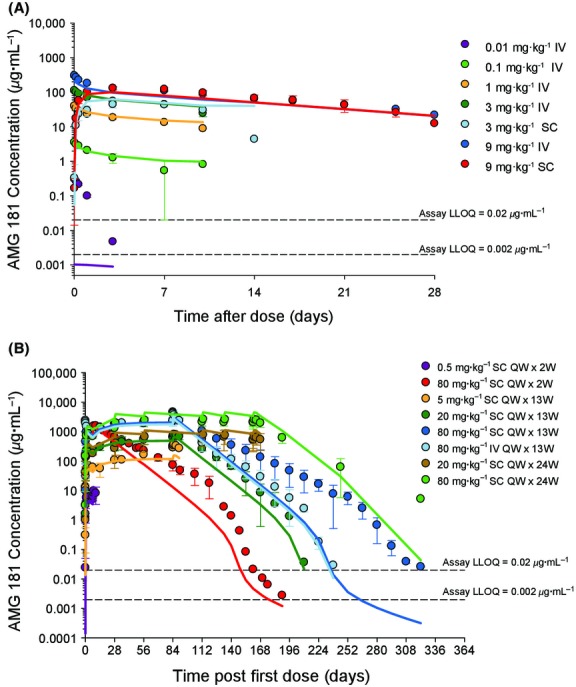
Simultaneous fitting of the individual AMG 181 concentration–time data from all cynomolgus monkeys using the two-compartment TMDD QE PK model: (A) Single IV or SC dose and (B) 2-weekly, 13-weekly, or 24-weekly IV or SC doses. Symbols represent mean (±SD) observations. Solid lines represent the mean of model predicted individual concentration–time profiles in the respective cohort.

**Figure 3:**
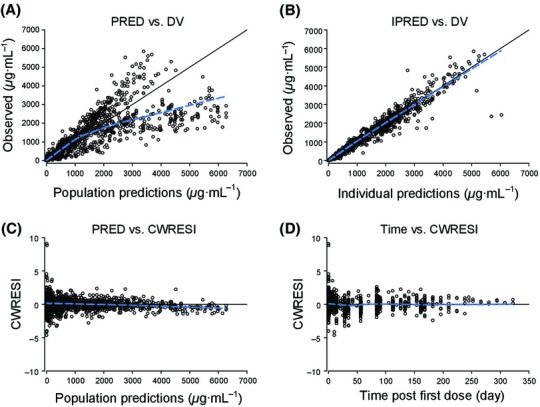
Diagnostic plots for the two-compartment TMDD QE PK model: (A) Observed versus population predicted concentrations. (B) Observed vs. individual Bayesian predicted concentrations. (C) Conditional weighted residuals with interactions versus population predicted concentrations. (D) Conditional weighted residuals with interactions versus time post first dose. Symbols are observations and the blue lines are LOESS regression (local regression) lines. The black lines in (A) and (B) are lines of unity, while, in (C) and (D), lines of zero CWRESI.

In the final model, the individual AMG 181 concentration–time data for all cynomolgus monkeys from Studies A–E were simultaneously fit using a two-compartment TMDD QE PK model (Fig.1). This model performed well for single and multiple dose administration with the exception of lowest single- dose cohort (0.01 mg·kg^−1^ IV), where the model underpredicted the observed concentrations (Fig.2). The diagnostic plots presented in Figure 3 suggest that the PK model characterized the observed concentration–time data well. Some divergence from the line of identity was observed at higher concentrations in the DV versus PRED plot. This deviation may have been due, in part, to the attempt to reconcile the PK from the 80 mg·kg^−1^ IV and SC doses, where SC dosing resulted in higher exposure compared to the IV dose (Fig. S1A).

Table 2 summarizes the AMG 181 population parameters and IIV in cynomolgus monkeys estimated in the final model. The population PK parameter estimates as well as the IIVs were generally well estimated with relative standard error (%SE) of less than 30%. In Studies A, C, and D, where AMG 181 was administered both SC and IV, relative bioavailability after SC was estimated to be 80–96% via noncompartmental analysis. In our first PK modeling attempt, the SC bioavailability (F1) was initially estimated to be close to 1 and was subsequently set to 1.0 (or 100%) to stabilize the model and to provide a conservative approach when the same assumption was also applied in prediction of human PK. No IIV on F1 was estimated.

**Table 2:** AMG 181 PK parameter estimates in cynomolgus monkeys through simultaneous fitting of the individual AMG 181 concentration- time data using the two-compartment pharmacokinetic target-mediated drug disposition (TMDD) model with quasi-equilibrium (QE) approximation

PK parameter (unit)	Estimate (%SE)	IIV (%SE)
*K*_A_ (day^−1^)	0.846 (3.8)	NE
CL (mL·day^−1^)	12.9 (5.9)	44.1 (20)
*V*_c_ (mL)	116 (7.9)	46.1 (17)
K_12_ (day^−1^)	0.617 (13)	Fixed to 0
K_21_ (day^−1^)	0.877 (8.8)	Fixed to 0
*K*_d_ (nmol^*^L^−1^)	Fixed to 0.013	Fixed to 0
*R*_tot_ (nmol^*^L^−1^)	9.18 (20)	180 (23)
*K*_int_ (day^−1^)	0.0215 (6.0)	Fixed to 0
Correlation CL, *V*_c_	0.642 (27)	
Proportional residual error (% CV)	20.1 (1.9)	
Additive residual error SD (nmol^*^L^−1^)	1.11 (14)	

CV, coefficient of variation; IIV, interindividual variability; NE, not estimated.

The estimated population central volume of distribution (*V*_c_) was 116 mL, which is physiologically plausible for a typical 2.8 kg cynomolgus monkey (Davies and Morris 1993). Based on the typical PK parameters, the linear elimination half-life of AMG 181 was estimated to be 12 days. Since the target (*α*_4_*β*_7_ receptor) concentrations were not available and few drug concentrations were measured at the terminal phase, *R*_tot_ and *K*_d_ could not be simultaneously estimated. Therefore, *K*_d_ was fixed to 0.013 nmol/L, the EC_50_ value of AMG 181 binding to *α*_4_*β*_7_ receptors on monkey primary T cells in vitro, in the final model and *R*_tot_ was estimated relative to this assumption (Table 2).

**Figure 4:**
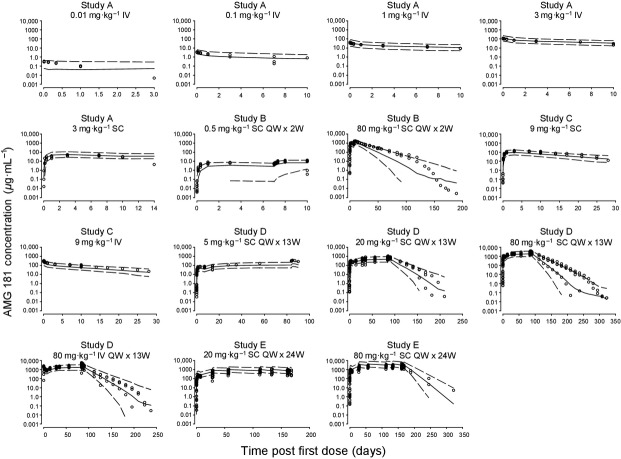
Visual predictive checks for the two-compartment TMDD QE PK model (by cohort): median predictions (solid lines) with 80% confidence intervals (10th and 90th percentiles; dashed lines). Symbols represent observed individual AMG 181 concentrations.

Figure 4 shows the internal model validation carried out using visual predictive checks (VPC). With 400 datasets simulated based on final parameter estimates, the estimated medians and the two-sided 80% prediction intervals (10th, 50th, and 90th percentiles) of the simulated AMG 181 concentration–time profiles contained the observed PK data well, especially in light of the small sample sizes per dose group and over an 8000-fold dose range (0.01–80 mg·kg^−1^).

### Modeling of AMG 181 PD in cynomolgus monkeys

**Figure 5:**
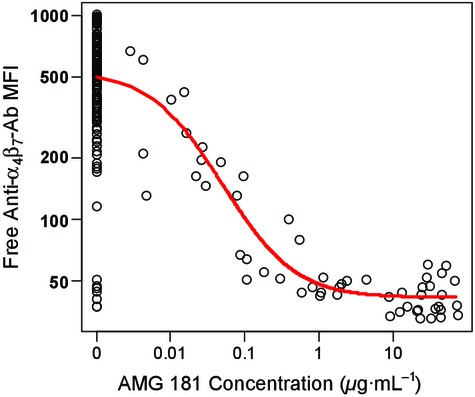
Individual observed free *α*_4_*β*_7_ receptor on CD4^+^ T cells (expressed as mean fluorescence intensity (MFI) of the detecting antibody for the free *α*_4_*β*_7_ receptor) versus AMG 181 concentrations (symbol). The line represents the *E*_max_ PD model fitting of the observed data.

For Study A, individual observed free *α*_4_*β*_7_ receptor on total CD4^+^ T cells (measured as MFI) versus AMG 181 concentration data is compared to the model fit in Figure 5. The estimated PD model parameters were 531 (MFI), 0.922, and 0.0140 ± 0.0082 *μ*g·mL^−1^ for *E*_0_, *E*_max_, and EC_50_, respectively. Based on the model, the calculated values for EC_75_, EC_90_, and EC_99_ are 0.042, 0.126, and 1.38 *μ*g·mL^−1^, respectively.

As a pharmacologic consequence of *α*_4_*β*_7_ occupancy, counts of total CD4^+^ T cell and its subsets, including naïve and central memory cell counts, were elevated due to the inhibition of their trafficking into the intestinal tissues. Because the CD4^+^ T cell and its subset count data were sparse and highly variable in all studies, PD modeling attempts using cell count measures were not successful: qualitative assessments have been reported elsewhere (Pan et al. 2013).

### Allometric scaling, simulation, and dose selection in humans

**Figure 6:**
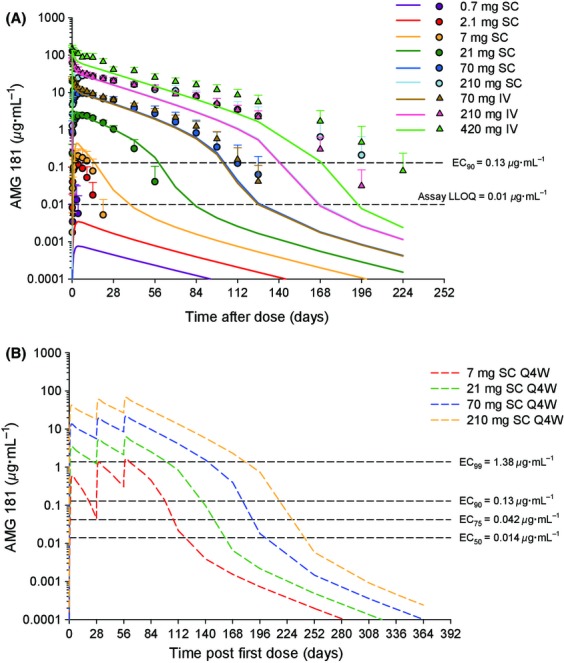
The two-compartment TMDD QE PK model predicted typical AMG 181 PK profiles in humans: (A) Single SC or IV dose (lines) overlaid with the observed mean (SD) data (symbols). (B) Three-monthly SC doses. *E*_max_ PD model predicted EC_50_, EC_75_, EC_90_, and EC_99_ values are presented to illustrate duration of AMG 181 concentration coverage over *α*_4_*β*_7_ receptor under each dosing regimen.

The estimated AMG 181 PK parameters in humans, determined through allometric scaling from parameters determined in cynomolgus monkeys, are presented in Table 3. These PK parameters (e.g., CL and *V*_c_) are physiologically reasonable for kinetics of monoclonal antibodies in human (Davies and Morris 1993). Using these parameters, the AMG 181 concentration–time profiles were simulated under various single SC or IV dosing regimens or multiple SC dosing regimens (Fig.6). The EC_50_, EC_75_, EC_90_, and EC_99_ values for AMG 181 occupancy of free *α*_4_*β*_7_ receptors on CD4^+^ T cell in humans were assumed to be the same as those estimated in monkeys and are given as reference concentration levels for the pharmacologic effect in Figure 6A. These levels were used to assess the duration of human AMG 181 concentration over various target saturation levels in designing the subsequent FIH study.

**Table 3:** Estimated human AMG 181 pharmacokinetic parameters. The PK parameters CL, *V*_c_, K_12_, and K_21_ were allometric scaled from cynomolgus monkeys. F1, *K*_d_, *K*_int_, and *R*_tot_ were assumed to be the same as the cynomolgus monkeys’ parameters

PK parameter (unit)	Human estimate
F1	1.0 (NS)
*K*_A_ (day^−1^)	0.846 (NS)
CL (mL·day^−1^)	144
*V*_c_ (mL)	2900
K_12_ (day^−1^)	0.276
K_21_ (day^−1^)	0.392
*K*_d_ (nmol^*^L^−1^)	0.013 (NS)
*R*_tot_ (nmol^*^L^−1^)	9.18 (NS)
*K*_int_ (day^−1^)	0.0215 (NS)

NA, not applicable; NS, not scaled.

Based on our simulations, the estimated *C*_max_ at the starting dose of 0.7 mg SC would be associated with approximately 10% of the maximum PD effect (EC_10_), while the 21, 70, 210, and 420 mg doses were predicted to cover EC_90_ for approximately 2, 3, 5, and 6 months, respectively (Fig.6A). All four multiple dosing regimens were predicted to maintain AMG 181 concentration above EC_90_ for 3–8 months (Fig.6B).

The model-predicted AMG 181 *C*_max_ and AUC values in humans, based on the simulated PK profiles, are presented in Figure 6, and the estimated exposure margins relative to the NOAEL of 80 mg·kg^−1^ in cynomolgus monkeys are listed in Table 4. Based on these exposure ratios, a greater than 490,000-fold safety margin at the starting dose of 0.7 mg SC in healthy subjects was anticipated. This low starting dose of 0.7 mg was selected because it was predicted to only briefly cover the predicted EC_10_, in accordance with the Minimal Anticipated Biological Effect Level (MABEL, EMA 2007; Lowe et al. 2010; Yu et al. 2011) guidance by the European Medicines Agency (EMA). Also, based on the US FDA guidance on “Estimating the Maximum Safe Starting Dose in Initial Clinical Trials for Therapeutics in Adult Healthy Volunteers” (FDA 2005), the starting dose of 0.7 mg was approximately 1/260th of the calculated maximum recommended safe starting dose (MRSD). The MRSD was calculated to be 2.58 mg·kg^−1^ based on the body surface scaling with exponent 0.67, with an average body weight of 3 kg for monkeys and 60 kg (per MRSD FDA guidance) for humans, a NOAEL of 80 mg·kg^−1^ in monkeys, and a default safety factor of 10.

**Table 4:** Predicted AMG 181 pharmacokinetic exposure parameters in humans based on the two-compartment model with target-mediated drug disposition (TMDD) and quasi-equilibrium (QE) approximation

Proposed human dose			PK parameter		Exposure ratio	
Dose (mg)	Dose (mg·kg^−1^)	Route	*C*_max_ (*μ*g·mL^−1^)	AUC_0-inf_ or AUC_tau_ (*μ*g·h·mL^−1^)	*C*_max_	AUC_0-inf_
Single ascending dose study						
0.7	0.01	SC	0.0009	0.985	5520000	492000
2.1	0.03	SC	0.004	3.81	1120000	127000
7	0.1	SC	0.604	186	8030	2610
21	0.3	SC	3.48	1940	1390	250
70	1	SC	13.5	9500	358	51
210	3	SC	42.4	32,300	114	15
70	1	IV	22.7	9520	214	51
210	3	IV	70.8	32,300	69	15
420	6	IV	143	67,000	34	7
Multiple ascending dose study (Q4W × 3): after the 3rd dose						
7	0.1	SC	1.69	782	2870	620
21	0.3	SC	6.29	4120	771	118
70	1	SC	22.4	17,100	217	28
210	3	SC	68.4	55,300	71	9

The exposure ratios (margins) were calculated based on NOAEL of 80 mg·kg^−1^ in cynomolgus monkeys (AUC_tau_: AUC within the 3rd dosing interval for the ascending multiple dose study).

### Comparison of predicted versus observed AMG 181 PK in humans

**Figure 7:**
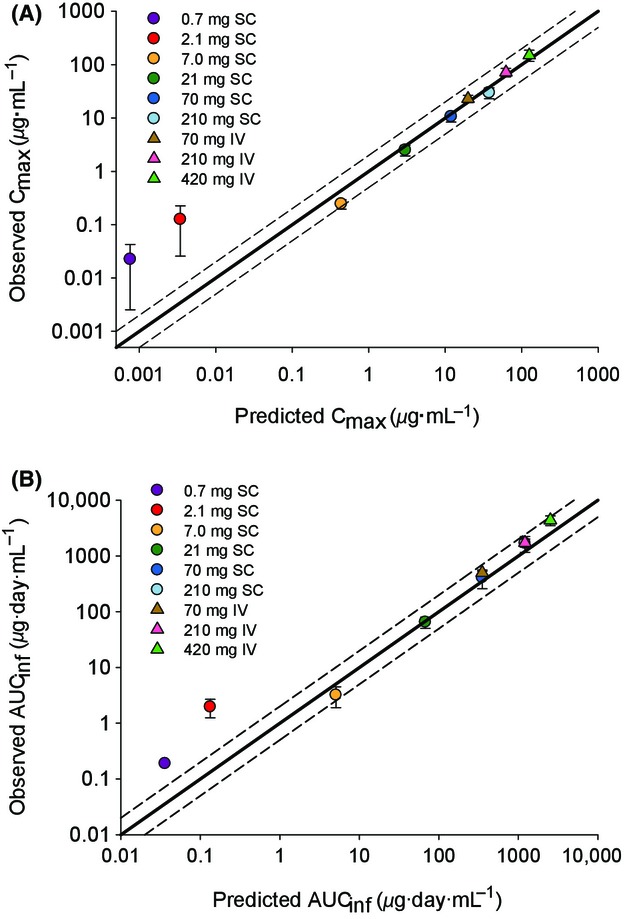
The mean (SD) observed AMG 181 *C*_max_ (A) and AUC_inf_ (B) data versus the values calculated using the two-compartment TMDD QE PK model predictions in humans after single SC or IV dose. The middle lines represent the lines of unity, while the upper and lower lines represent the lines of ±2-fold of unity.

The observed AMG 181 *C*_max_ and AUC_inf_ values in the FIH study were well predicted based on scaling of cynomolgus monkey PK (Fig.7). With the exception of lower doses (0.7 and 2.1 mg AMG 181), where *C*_max_ and AUC_inf_ were underpredicted, the observed human AMG 181 *C*_max_ and AUC_inf_ values were within twofold of the predicted values, consistent with what has previously been reported for allometric scaling of monoclonal antibody PK from cynomolgus monkey to human (Dong et al. 2011). Given the large exposure margins at the proposed FIH starting dose of 0.7 mg, this slight underprediction was not expected to have consequences for patient safety.

## Discussion and Conclusions

AMG 181 is a human monoclonal antibody that specifically targets intestinal-homing T cells and has been tested for pharmacological activity in vitro as well as for safety and PK/PD in vivo in the pharmacologically relevant species *M. fascicularis* (cynomolgus monkey) (Pan et al. 2014). This report presents a quantitative translational approach for the selection of a safe starting dose and pharmacologically relevant single and multiple dose escalation schemes for Phase 1 clinical trials in healthy volunteers and IBD subjects.

Based on visual inspection of the PK profiles in monkeys, AMG 181 disposition was dose- and concentration- dependent with typical linear clearance dominating at higher concentrations and nonlinear clearance dominating at low concentrations (<1 *μ*g·mL^−1^, ∼7 nmol/L), likely due to saturable binding of AMG 181 to the target T-cell surface *α*_4_*β*_7_ receptors. To describe the parallel linear and nonlinear disposition, a TMDD model (Mager and Jusko 2001) was utilized and all individual monkey AMG 181 concentration–time data were fitted simultaneously.

AMG 181 binds to *α*_4_*β*_7_ receptor with high affinity with a dissociation constant (*K*_D_ = *K*_off_/*K*_on_) of 13 pmol*L^−1^, suggesting that the drug–target association is faster than drug dissociation/distribution/elimination as well as elimination of the target and drug–target complex. In this case, the QSS approximation to TMDD, which assumes that drug–target complex rapidly approaches a quasi-steady state, is reasonable (Gibiansky and Gibiansky 2009). To overcome the difficulties with model parameter identifiability during model development, QSS model was further simplified to a QE model with *K*_ss_ = *K*_d_ = *k*_off_/*k*_on_.

During the model development, fitting of an empirical two-compartment PK model with parallel linear and nonlinear Michaelis–Menten (M-M) (Dong et al. 2011) elimination was also attempted. The M-M model fit similarly well to the high concentration data as did the TMDD models, but underpredicted concentrations at the terminal elimination phase where AMG 181 concentration were <0.1 *μ*g*mL^−1^ (Fig. S3A and B); consistent with what has been demonstrated by Gibiansky and Gibiansky (2009), Yan et al. (2010). The exception to this was for the lowest dose (0.01 mg·kg^−1^ IV) where, unexpectedly, the M-M model provided a better model fit than QE TMDD model. For our purpose, selection of either M-M model or QE TMDD model would have been sufficient to inform selection of FIH dosing. The TMDD QE model was chosen for further analyses and human dose predictions due to better overall model fit at clinically relevant doses.

Three factors posed challenges for better estimation of the AMG 181 PK across all concentrations and dose routes. First, AMG 181 is a human antibody and would unavoidably be immunogenic in monkeys, especially at lower concentrations. Second, only a limited number of monkeys were assigned to the recovery phase of the GLP studies. Both factors reduced the potential amount of PK data available for modeling the terminal elimination phase. Third, the relatively higher exposures observed after SC administration of 80 mg·kg^−1^ AMG 181 than after IV administration of the same dose (Studies B and D vs. Study D, Fig.2B) was undoubtedly a challenge for the model to reconcile. While this could potentially by explained by PK variability and small sample sizes, the observed differences could also have stemmed from nonneutralizing AMG 181 immune complex formation, which might have served as a reservoir (Chirmule et al. 2012) for AMG 181 through gradual disassociation and slow release of AMG 181 during the terminal elimination phase. The estimated slow half-life of ∼32 days (= 0.693/*K*_int_) for *α*_4_*β*_7_ receptor internalization may support this hypothesis and thus *K*_int_ might be a measure of both target receptor internalization and immune complex elimination.

The immunogenic response against AMG 181 clearly impacted specific PK data points, as illustrated for two representative animals in Figure S2. For this reason, 120 ADA-positive concentration–time points (representing less than 7% of total data) were excluded from model. This approach is conservative from a safety perspective as it resulted in higher estimated exposures in monkey and consequently in human. Additionally, since AMG 181 is a human IgG2, it is anticipated to carry less immunogenic potential in humans. Indeed, no ADAs were reported in the Ph1a study (Pan et al. 2014).

Dong et al. (2011) suggest that translation of nonlinear elimination to human might be improved by accounting for the between-species difference in target expression and binding. The lack of knowledge of *α*4*β*7 receptor abundance in cynomolgus monkeys and human necessitated the assumption that AMG 181 has the same in vivo pharmacology characteristics and *α*_4_*β*_7_ receptor abundance in cynomolgus monkeys and humans. This assumption was justified based on similar in vitro binding to primary T cells and blocking of *α*_4_*β*_7_:MAdCAM-1 binding on primary T cells and T-cell adhesion for cynomolgus monkey and human (Pan et al. 2013). Additionally, potential differences are not expected to affect patient safety due to the large observed safety margins.

The 75% and 90% receptor occupancy levels for natalizumab (anti-*α*_4_) and vedolizumab (anti-*α*_4_*β*_7_) have been reported to be related to the clinical efficacy in multiple sclerosis (FDA 2003) and IBD (Feagan et al. 2005, 2008). Therefore, the AMG 181 EC_75_ and EC_90_ values for *α*_4_*β*_7_ receptor occupancy were used as guides to aid the selection of AMG 181 dosing regimens in UC and CD clinical trials: 7, 21, 70, or 210 mg SC once every 1, 2, 3, or 4 months, respectively, would maintain AMG 181 concentration above EC_90_ within each dosing interval (Fig.6B) and thus should be efficacious.

Analysis of the available clinical PK, safety and efficacy data from the ongoing studies have shown consistency with our prediction results as shown in Figure 7 (Pan et al. 2014). The predictions of C_max_ and AUC were within twofold for single doses between 7 and 420 mg which is well accepted for large and small molecules (Hosea et al. 2009; Dong et al. 2011).

In conclusion, AMG 181 in vivo pharmacology from cynomolgus monkeys was successfully translated to humans using the described quantitative translation approach. The developed model was successfully employed to support the selection of a safe starting dose and pharmacologically as well as clinically relevant single and multiple dose escalation schemes for AMG 181 clinical trials in healthy volunteers and IBD subjects.

## Abbreviations

AUC: area under the concentration–time curve
CD: Crohn’s disease or cluster of differentiation
CHO: Chinese Hamster Ovary cells
CL: Clearance
*C*_max_: maximum observed concentration
EC_50_: concentration at half-maximal effect
ECL: electrochemiluminescence
F1: subcutaneous bioavailability
GLP: good laboratory practice
HEK: human embryonic kidney cells
IBD: inflammatory bowel diseases
IgG: immunoglobulin G
IIV: interindividual variability
IV: intravenous(ly)
JC virus: John Cunningham polyomavirus
*K*_A_: absorption rate constant
*K*_D_: dissociation constant
*K*_int_: internalization rate constant
*K*_m_: Michaelis–Menten constant
*K*_ss_: quasi-steady-state constant
LLOQ: lower limit of quantification
MAdCAM-1: mucosal addressin cell adhesion molecule 1
M-M: Michaelis–Menten
NIH: National Institutes of Health
PBMC: peripheral blood mononuclear cells
PD: pharmacodynamics(s)
PK: pharmacokinetic(s)
PML: progressive multifocal leukoencephalopathy
QE: quasi-equilibrium
QSS: quasi-steady state
*R*_tot_: total receptor concentration
SC: subcutaneous(ly)
TMDD: target-mediated drug disposition
UC: ulcerative colitis
*V*_c_: central volume of distribution
*V*_max_: maximum nonlinear elimination rate
VPC: visual predictive check

## Supporting Information

**Figure S1.** Diagnostic plots for the two-compartment TMDD QE PK model by study and cohort: (A) Observed versus population predicted concentrations. (B) Observed versus individual predicted concentrations. For study A, B, and C cohorts were combined for plotting purposes. **Figure S2**. Concentration-time profiles in two representative animals to illustrate the ADA effect on PK. Red symbols and lines represent the mean (±SD) of the same dose group, black symbols indicate data of an individual subject; ADA-positive data points are depicted by x. **Figure S3.** Simultaneous fit of the individual AMG 181 concentration-time data from all cynomolgus monkeys using the two-compartment MM PK model: (A) Single IV or SC dose and (B) 2-weekly, 13-weekly, or 24-weekly IV or SC doses. Symbols and dotted lines represent mean (±SD) observations. Solid lines represent the mean of model predicted individual concentration-time profiles in the respective cohort.
