# Supplementary material for: Prediction of clinical pharmacokinetics of AMG 181, a human anti-α4β7 monoclonal antibody for treating inflammatory bowel diseases
Source: Pharmacol Res Perspect. 2014 Dec 9;3(1):e00098. doi: 10.1002/prp2.98 (PMC4317229; doi:10.1002/prp2.98)
Supplement: Supplementary file 1 [file prp20003-e00098-sd1.docx]

**Supplemental Figure 1.** Diagnostic plots for the two-compartment TMDD QE PK model by study and cohort: (A) observed vs. population predicted concentrations. (B) Observed vs. individual predicted concentrations. For study A, B, and C cohorts were combined for plotting purposes.

| **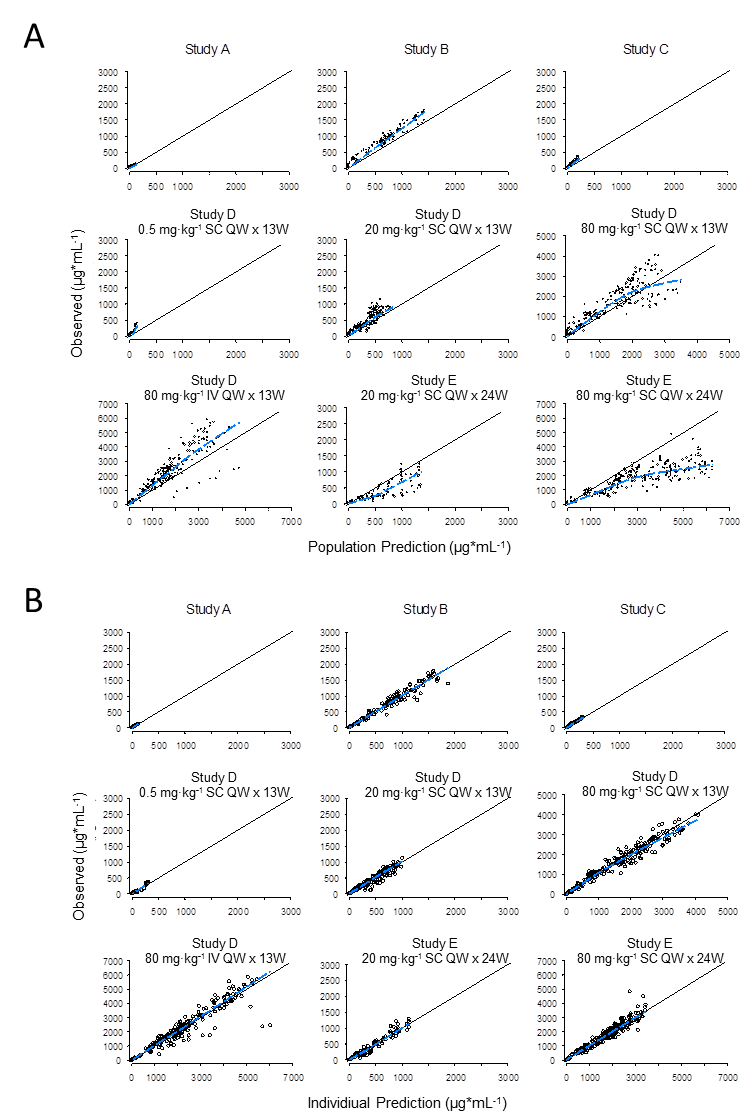** |
| --- |

**Supplemental Figure 2**. Concentration-time profiles in two representative animals to illustrate the ADA effect on PK. Red symbols and lines represent the mean (± SD) of the some dose group, black symbols indicate data of an individual subject; ADA-positive data points are depicted by x.

Time post First Dose (days)

Time post First Dose (days)

AMG 181 Concentration (µg*mL^-1^)

AMG 181 Concentration (µg*mL^-1^)

**Supplemental Figure 3.** Simultaneous fit of the individual AMG 181 concentration-time data from all cynomolgus monkeys using the two-compartment MM PK model: (A) Single IV or SC dose and (B) 2-weekly, 13-weekly, or 24-weekly IV or SC doses. Symbols and dotted lines represent mean (±SD) observations. Solid lines represent the mean of model predicted individual concentration-time profiles in the respective cohort.

**A**

**B**
